# STAYIN’ ALIVE: CAN VIRTUAL COLONOSCOPY SURVIVE AS A SCREENING METHOD?

**DOI:** 10.1590/S0004-2803.24612026-E01

**Published:** 2026-08-14

**Authors:** Pedro Averbach, Marcelo Averbach

**Affiliations:** 1 Instituto Sírio-Libanês de Ensino e Pesquisa, Cirurgiões colorretais, São Paulo, SP, Brasil.; 2 Fundadores da ONG Zoé, São Paulo, SP, Brasil.; 3 Universidade de São Paulo, Faculdade de Medicina, Disciplina de Técnica Cirúrgica e Cirurgia Experimental, São Paulo, SP, Brasil.

Computed tomography colonography (CTC), or “virtual colonoscopy”, uses low-dose multidetector CT to generate three-dimensional reconstructions of the colon and rectum. The technique can reveal polyps, cancers, and extracolonic findings without sedation and with minimal invasiveness. Yet, when lesions are detected, therapeutic colonoscopy remains necessary, creating an intrinsic limitation for population-based screening.

Two decades ago, enthusiasm for CTC was high. It promised to combine safety, patient comfort, and comprehensive visualization of the colon in a single, noninvasive test. Today, however, its role has become far less clear. Two recent large-scale studies - a 10-year national analysis from the United States (Abbas et al., 2024) and a 20-year single-center experience from the University of Wisconsin (Pickhardt et al., 2024) - provide a remarkably coherent picture of both the strengths and the decline of this modality.

The Wisconsin group reported 15,431 examinations in 11,830 patients over two decades, with 9,168 performed for primary screening. Among these, 15.9% detected polyps ≥6 mm, 5.7% had lesions 10-29 mm, and 0.6% revealed masses ≥3 cm. The referral rate for optical colonoscopy was 11.5%, and the positive predictive value for lesions ≥6 mm reached 91.6%. Histologic analysis confirmed adenocarcinoma in 0.2%, advanced adenoma in 4.0%, non-advanced adenoma in 3.4%, sessile serrated lesions in 0.5%, and hyperplastic polyps in 1.2%. No perforations or major complications occurred, and extracolonic findings prompted additional investigation in 6% of cases, including extracolonic malignancies (0.4%) and abdominal aortic aneurysms (0.3%). Yet the most striking result was the collapse in screening volume-from 1,589 studies in 2005 to only 72 in 2023, a 96% decline. The only indication that remained stable was evaluation after incomplete colonoscopy^1^.

On a national scale, Abbas et al. analyzed 58,058 adults in the U.S. National Health Interview Survey between 2010 and 2021. The prevalence of CTC use remained below 2% until 2019, rising modestly to 3.5% in 2021, coinciding with the COVID-19 pandemic and the need for socially distanced procedures. Utilization was higher among Black and Hispanic individuals, patients with lower income, and those with chronic diseases such as COPD, diabetes, or prior cancer-populations that historically face barriers to colonoscopy. These findings suggest that while CTC has not achieved mainstream acceptance, it may still contribute to reducing disparities in colorectal cancer screening where access to endoscopy or sedation is limited^2^.

Several factors explain the global retreat of CTC as a screening option:

1. Procedural limitations - bowel preparation is still required; lesions cannot be removed during the same session; and sensitivity remains lower for flat and serrated lesions, now recognized as responsible for many interval cancers.
2. Competitive advances - high-definition optical colonoscopy offers immediate diagnosis and therapy, while stool-based tests (FIT, FIT-DNA) gained wide acceptance for their low cost, simplicity, and scalability.
3. Systemic barriers - reimbursement and professional endorsement lagged; integration into primary-care workflows remained patchy; and many radiology centers lacked standardized quality programs.
4. Changing value calculus - patients and payors increasingly favor tests combining effectiveness, convenience, and definitive management in one encounter.

Even the incidental extracolonic findings that once seemed an advantage have proven to be both a virtue and a vice. While they can reveal clinically significant abnormalities, such as aneurysms or early-stage cancers, they often lead to additional imaging, anxiety, and costs. The balance between benefit and overdiagnosis remains debated and affects the cost-effectiveness of the method.

In Brazil, CT colonography never achieved widespread use. Its practice is largely restricted to major private diagnostic centers and a few academic hospitals, usually as a second-line option after incomplete colonoscopy or when endoscopy is contraindicated. Within the public health system (SUS), the exam is rarely performed due to limited access to appropriate software, trained radiologists, and clear reimbursement pathways. Unlike the United States, where the decline of CTC marks the end of a long trajectory, in Brazil the method never truly became mainstream. Yet, where available and well executed, it can still be valuable-particularly when integrated into broader colorectal cancer prevention strategies^3^.

CT colonography adds value in a few specific contexts:

- when colonoscopy is incomplete or technically impossible;
- when patients refuse or have contraindications to endoscopy or sedation;
- when endoscopic capacity is limited or waiting lists are long;
- when extracolonic evaluation may offer additional clinical insight; and
- when teams are adequately trained and quality standards are ensured.

The challenge in Brazil is not revival but strategic incorporation-ensuring availability where it genuinely benefits patients while fostering local expertise to support safe, high-quality execution. Nevertheless, CTC is becoming a victim of its own narrowing niche. In Brazil and elsewhere, the exam is now confined to very specific indications, resulting in low procedural volumes that undermine the maintenance of professional proficiency. Even when CTC is indicated, few specialists are sufficiently trained to perform and interpret it confidently. Without structured training programs, reference centers, or consistent reimbursement, the method risks fading into obsolescence-not because it lacks diagnostic accuracy, but because it no longer fits the logistical, economic, and educational realities of modern colorectal cancer screening.

The future of CTC, therefore, appears less as a story of resurgence than of gradual marginalization. It may persist as a residual tool-useful in select cases but increasingly overshadowed by more accessible, cost-effective, and integrated screening approaches. Unless deliberate efforts are made to preserve expertise and ensure technical quality, its disappearance from daily practice may pass unnoticed-not as a failure of technology, but as the quiet fading of a once-promising idea whose time has, perhaps, already passed.

## Data Availability

data-available-upon-request
